# Perceptions of the impact of COVID-19 in Tennessee, USA: a retrospective study

**DOI:** 10.7717/peerj.15473

**Published:** 2023-07-10

**Authors:** Tamara L. Chavez-Lindell, Katie A. Cahill, Kristina W. Kintziger, Agricola Odoi

**Affiliations:** 1 Biomedical and Diagnostic Sciences, University of Tennessee, Knoxville, United States of America; 2 Howard H. Baker Jr. Center for Public Policy, University of Tennessee, Knoxville, United States of America; 3 Department of Public Health, University of Tennessee, Knoxville, United States of America

**Keywords:** COVID-19, Tennessee, USA, Impact, Questionnaire survey, Retrospective, Cross-sectional study, Logistic regression, Perceptions

## Abstract

**Background:**

Despite high incidence and mortality risks associated with COVID-19 during the pandemic, stay-at-home orders and vaccination recommendations were met with varying levels of acceptance in Tennessee. Understanding perceptions of individuals regarding the health and economic impacts of COVID-19 is necessary to address public concerns while ensuring appropriate public health response. Therefore, the objectives of this study were to (a) investigate differences in opinions among residents of Tennessee regarding the impacts of COVID-19; and (b) identify socioeconomic and demographic predictors/determinants of these opinions.

**Methods:**

This retrospective cross-sectional study was conducted using survey data collected in nine waves during 2020. Distributions of survey-weighted sociodemographic characteristics and respondent perceptions of the impact of COVID-19 were computed. Weighted logistic models were used to investigate predictors of a number of perceptions: whether the health or economic impact was greater, concern for respondent’s health, concern for family’s health, and willingness to accept COVID-19 vaccine.

**Results:**

The study included a total of 9,754 survey respondents. Approximately equal percentages considered COVID-19 to have a greater economic (48.4%) versus health impact (51.6%). Just 40.1% of the respondents reported that they would definitely accept a COVID-19 vaccine. Age group, race, educational attainment, and household composition were significant (*p* < 0.05) predictors of all investigated perceptions regarding COVID-19. Lack of prior infection was the strongest predictor of the perception of COVID-19 having a greater impact on health (OR = 2.40, *p* < 0.001), concern for respondent’s health (OR = 1.86, *p* = 0.002), and concern for family members’ health (OR = 1.90, *p* = 0.001). Compared to males, females had higher odds of identifying the health impact of COVID-19 as greater (OR = 1.09, *p* = 0.041) and reporting concern for family health (OR = 1.14, *p* = 0.003). However, they had lower odds (OR = 0.63, *p* < 0.001) of willingness to accept vaccine than males.

**Conclusion:**

These findings improve our understanding of the drivers of health behaviors, including vaccine hesitancy, and are useful for guiding public health outreach/education programs.

## Background

The coronavirus disease (COVID-19) pandemic caused by the SARS-CoV-2 virus has led to severe economic and social disruptions worldwide. Within a month of the disease being declared a global pandemic by the World Health Organization (WHO) (Cucinotta & Vanelli, 2020), the governors of multiple US states, including Tennessee, issued stay-at-home orders, requiring residents to remain at home except for essential workers and essential needs shopping (Moreland et al., 2020; Office of the Governor of Tennessee, 2020). The stay-at-home orders were aimed at reducing the risk of community spread and, subsequently, the burden on healthcare facilities (Centers for Disease Control and Prevention, 2021). However, these orders have also had negative socioeconomic impacts (Lou, Shen & Niemeier, 2020; Chang et al., 2021) and have been met with varying levels of compliance (Hamidi & Zandiatashbar, 2021; Brodeur, Grigoryeva & Kattan, 2021). Understanding the perceptions of different communities regarding the health and economic impacts of COVID-19 is necessary to effectively address public concerns while ensuring appropriate public health response to control the pandemic.

Tennessee is a largely rural state and has experienced significant health, economic, and social impacts associated with COVID-19 (Sycamore Institute, 2021). Since the pandemic began to spread throughout the United States, COVID-19 incidence and mortality risks in Tennessee have remained among the highest in the nation, reaching 29,602 cases per 100,000 residents by early 2022 (Centers for Disease Control and Prevention, 2022). Accordingly, the pandemic has impacted the health, social, and economic wellbeing of Tennesseans. Between April 2019 and April 2020, there was a substantial decline in employment in Tennessee (Department of Labor and Workforce Development, 2020). The precipitous decrease in economic stability compared to other states across the Southeast (US Bureau of Labor Statistics, 2022a; US Bureau of Labor Statistics, 2022b) may have further compounded the challenges faced by many families during the pandemic and reinforced entrenched health disparities experienced across rural/urban (Perry, Aronson & Pescosolido, 2021; Ullrich & Mueller, 2022) as well as racial/ethnic groups (Fortuna et al., 2020; Hawkins, Charles & Mehaffey, 2020; Acosta et al., 2021). The pandemic has also significantly impacted mental health across the nation (Kämpfen et al., 2020). Provisional data indicate that psychological reactions to the COVID-19 pandemic have been widespread, including symptoms of anxiety, depression, and self-reported stress (Kämpfen et al., 2020; Rajkumar, 2020). Mental health impacts of the pandemic have been disproportionately higher among communities of color (Saltzman et al., 2021) and rural areas (Mueller et al., 2020), compared to other groups. Therefore, the objective of this study was to investigate regional (rural/urban), socioeconomic, and demographic differences in the opinions of Tennessee residents regarding the impacts of COVID-19 and identify predictors of these opinions.

## Methods

### Ethics approval

This study was reviewed and approved by the University of Tennessee IRB (IRB Number: UTK IRB-20-05970). The IRB assessment determined that this study does not involve Human Subjects as defined in 45 CFR 46.102 (e) (1), because it did not involve obtaining information by intervention or interaction with living individuals, nor did it obtain identifiable private information about living individuals. Thus, the IRB assessment determined that neither review nor certification of exemption from IRB review was needed. This implies no consent was necessary. All study methods were carried out in accordance with relevant guidelines and regulations. The study used anonymized secondary data provided to the investigators in such a manner that the identity of human subjects could not be ascertained directly or through identifiers linked to the subjects. The investigators did not contact the subjects and did not re-identify subjects.

### Design, setting, and data source

This retrospective cross-sectional study used the Tennessee Pulse survey data that included responses from 9,754 participants from across the state of Tennessee. The Tennessee Pulse (TN-Pulse) survey was part of a collaborative effort between the Tennessee Economic Recovery Group, the University of Tennessee’s Howard H. Baker Jr. Center for Public Policy, and the University of Tennessee’s Social Work Office of Research and Public Service to capture the attitudes and consumer sentiments of Tennessee residents toward COVID-19. The survey collected data in 12 waves between May 2020 and March 2021. Waves 1–9 included in the present analysis correspond to those surveys conducted between May and December 2020. The TN-Pulse survey was conducted through a panel survey which pre-screened respondents to collect respondent demographics, permitting close adherence to the proportional sampling strategy. Black respondents were over-sampled to ensure adequate representation, with *post hoc* weighting subsequently used to balance the total sample as each additional wave became available.

### Variable selection and data management

Four outcome variables reflecting respondent perceptions of the impact of COVID-19 were selected from the TN-Pulse survey instrument. These included whether the health impact or economic impact of COVID-19 was greater, concern for their own health and wellbeing, concern for their family’s health and wellbeing, and whether the respondent would accept a free COVID-19 vaccine when available.

Data management was performed in STATA Version 16.1 (StataCorp, 2019). A dichotomous outcome variable, whether the health or economic impact of COVID-19 was greater, was retained as collected. Outcome variables reflecting participant concerns (concern for their own health and concern for their family’s health) were each re-coded from a 5-point scale (1 = Extremely concerned, 2 = Very concerned, 3 = Somewhat concerned, 4 = Not very concerned, 5 = Not at all concerned) to a dichotomous scale (0 = Low/Medium (Not very/Not at all concerned/Somewhat concerned), 1 = High (Extremely/Very concerned)). The variable assessing whether the respondent would accept a free COVID-19 vaccine (1 = Definitely would, 2 = Probably would, 3 = Probably would not, 4 = Definitely would not, 5 = Not sure), was also re-coded to a dichotomous scale (0 = Probably would not/Definitely would not, 1 = Not sure/Probably would/Definitely would). An additional variable, self-reported concern about contracting COVID-19, was recoded from a 6-point scale (1 = Extremely concerned, 2 = Very concerned, 3 = Somewhat concerned, 4 = Not very concerned, 5 = Not at all concerned, 6 = I have already tested positive for COVID-19) into two separate variables: concern about contracting COVID-19 (1 = Low (Not very/Not at all concerned), 2 = Medium (Somewhat concerned), 3 = High (Extremely/Very concerned)) and infection status (0 = no history of COVID-19, 1 = already tested positive for COVID-19), with the latter variable used in development of the predictive models.

Potential predictors of each outcome were selected based on biological plausibility of their association with the outcomes. A total of 16 potential predictors were considered for investigation: age group (18–24, 25–44, 45–64, 65+), sex, race (White yes/no, Black yes/no, Asian yes/no, American Indian/Alaskan Native yes/no, Hispanic/Latino yes/no), region (rural *vs.* metro county), educational attainment, employment status, household income, relationship status, children in household, older adults in household, COVID-19 infection status, and study wave. Response levels for most (11/16) categorical predictor variables (sex, race, region, children in household, older adults in household, educational attainment, study wave) were retained as collected. However, some of the categorical variables (age, employment status, relationship status, and annual household income) were re-coded as shown in Table 1.

**Table 1:** Original and re-coded variables utilized in the investigation of predictors of perceived COVID-19 impact, Tennessee.

Predictor Variables				
	Original Variable		Re-coded Variable	
		**Age group**		**Age group**
	1	18–24	1	18–24
	2	25–34	2	25–44
	3	35–44	3	45–64
	4	45–54	4	65+
	5	55–64		
	6	65+		
	**Employment status**		**Employment status**	
	1	Work full-time	1	Work full-time
	2	Work part-time	2	Work part-time
	3	Unemployed	3	Unemployed/Looking for work
	4	Looking for work	4	Student
	5	Student	5	Retired
	6	Retired	6	Caregiver
	7	Stay-at-Home Caregiver		
	**Relationship status**		**Relationship status**	
	1	Married	1	Married/Living with Someone
	2	Divorced	2	Divorced/Separated
	3	Separated	3	Dating/Single
	4	Living with someone		
	5	Dating		
	6	Single		
	**Annual household income**		**Annual household income**	
	1	Less than $15,000	0	Less than $50,000
	2	$15,000–$24,999	1	$50,000 or more
	3	$25,000–$34,999		
	4	$35,000–$49,999		
	5	$50,000–$74,999		
	6	$75,000–$99,999		
	7	$100,000–$149,999		
	8	$150,000 or more		
**Outcome Variables**				
	**Health *vs* Economic Impact**		**Health *vs* Economic Impact**	
	0	Economic Impacts	0	Economic Impacts
	1	Health Impacts	1	Health Impacts
	**Concern about own health**		**Concern about own health**	
	1	Extremely concerned	0	Low/Medium
	2	Very concerned	1	High
	3	Somewhat concerned		
	4	Not very concerned		
	5	Not at all concerned		
	**Concern about family’s health**		**Concern about family’s health**	
	1	Extremely concerned	0	Low/Medium
	2	Very concerned	1	High
	3	Somewhat concerned		
	4	Not very concerned		
	5	Not at all concerned		
	**Willingness to get vaccine**		**Willingness to get vaccine**	
	1	Definitely would	0	Probably Would Not/Definitely Would Not
	2	Probably would	1	Not Sure/Probably Would/ Definitely Would
	3	Probably would not		
	4	Definitely would not		
	5	Not sure		

### Descriptive analysis

All statistical analyses were performed using STATA Version 16.1 (StataCorp, 2019). Responses were weighted *post hoc* by race, gender, age, and rural/urban region of residence to ensure representativeness of the state’s population. All statistical analyses were performed using the statewide analytical weight variable (stateweight_overall) which balanced over-sampling of Black respondents to ensure adequate representation, particularly in the rural counties. Distribution of the categorical sociodemographic variables and their 95% confidence intervals were computed.

### Investigation of predictors

Analytical weights were used to build multivariable binary logistic models to investigate predictors of each of the outcome variables reflecting respondent perceptions of the impact of COVID-19 (Table 1). The models were built in two steps. First, univariable binary logistic models were used to assess the associations between the dichotomous outcomes and each of the potential predictors using the STATA xi: glm command. Statistical significance was assessed for all variables using the adjusted Wald test. Potential predictor variables with *p* ≤ 0.10 were considered for inclusion in the multivariable models for each outcome in the next step.

Next, multivariable models were built for each outcome using a manual backwards elimination process, sequentially removing those variables which did not have a significant (alpha level of 0.05) Wald test. The coefficients of all variables were reviewed at each step for evidence of confounding. Where removal of a variable resulted in a change of 20% or more in the coefficients of any of the other variables in the model, the removed variable was considered a confounder and retained in the model, regardless of its statistical significance. No biologically plausible two-way interactions were identified for assessment. Odds ratios (OR) and their 95% confidence intervals (CI) were computed for all variables retained in the final models.

## Results

### Descriptive analysis

The study included a total of 9,754 survey respondents. Respondents included a wide range of Tennesseans, 52% of whom were female (Table 2). Age was non-normally distributed (*p* < 0.001) and respondents ranged from 18 to 99 years, with a median age of 47 (Interquartile range: 32, 63). Most respondents were White, non-Hispanic (74.1%) and more than half (55.4%) were married or living with a partner. Half of respondents were employed full-time (39.8%) or part-time (11.4%), while 14.8% were unemployed. Educational attainment was approximately evenly distributed across levels at or above high school, with the largest percentage (35.4%) having attended some college. More than half (58.0%) of the respondents reported an annual household income of less than $50,000, which is below the state median of $53.320 (US Census Bureau, 2019). Households including children under age 18 were reported by 30.8% of respondents, while households including adults over the age of 50 were reported by nearly half (47.2%) of respondents (Table 2).

**Table 2:** Sociodemographic characteristics of respondents in a survey of perceived COVID-19 impact, Tennessee.

**Characteristic**	**Weighted frequency**	**Weighted percent**	**Weighted** 95% CI^*^
**Region**			
6-County Metro Region	3,807	39.1	19.5, 63.0
89-County Rural Region	5,933	60.9	37.0, 80.6
**Gender**			
Female	5,060	52.0	50.2, 53.7
Male	4,680	48.0	46.3, 49.8
**Age group**			
18–24	1,148	11.8	10.0, 13.9
25–44	3,261	33.5	31.1, 36.0
45–64	3,286	33.7	31.3, 36.3
65+	2,046	21.0	18.0, 24.4
**Race/Ethnicity**			
White Non-Hispanic	7,215	74.1	73.2, 74.9
Black Non-Hispanic	1,648	16.9	16.2, 17.7
Asian/Pacific Islander Non-Hispanic	151	1.6	1.3, 1.8
American Indian/Alaskan Native Non-Hispanic	120	1.2	1.0, 1.5
Other/Multiple Non-Hispanic	283	2.9	2.6, 3.3
Hispanic/Latino, Any Race	195	2.0	1.7, 2.3
**Marital status**			
Married/living with someone	5,398	55.4	50.9, 59.8
Divorced/separated	1,500	15.4	14.1, 16.8
Dating/single	2,842	29.2	24.8, 34.0
**Employment status**			
Work full-time	3,881	39.8	37.3, 42.5
Work part-time	1,110	11.4	10.3, 12.6
Unemployed/looking for work	1,441	14.8	13.3, 16.4
Student	365	3.8	3.0, 4.7
Retired	2,293	23.5	20.0, 27.5
At-home caregiver	633	6.5	5.5, 7.7
**Educational attainment**			
Less than high school	461	4.7	4.1, 5.5
High school graduate	2,793	28.7	25.6, 32.0
Some college/Associate’s degree	3,445	35.4	33.5, 37.3
Bachelor’s degree of higher	3,040	31.2	28.3, 34.3
**Annual household income**			
Less than $50,000	5,653	58.0	55.3, 60.7
$50,000 or more	4,087	42.0	39.3, 44.7
**Household members**			
Children under age of 18 living in home	3,004	30.8	29.0, 32.7
Adults over age of 50 living in home	4,595	47.2	44.0, 50.4
**Infection status**			
Previously infected	114	1.2	1.0, 1.4

**Notes.**

* CI, confidence interval.

Respondents’ opinions were approximately equally split regarding whether COVID-19 had a greater economic impact (48.4%) or health impact (51.6%) on their families (Table 3). More than half (55.5%) of respondents reported low or medium levels of concern about contracting COVID-19, while far fewer (43.3%) reported high levels of concern, and just 1.2% of respondents reported having already been infected with the virus. Respondents were much more concerned for the health of their family members, with almost two-thirds (61.6%) reporting high levels of concern. Similarly, 74.7% of respondents reported high levels of concern regarding the impact of COVID-19 on the United States. Reflective of the broad levels of concern reported, just 15.3% of respondents reported that they would definitely not accept a free COVID-19 vaccine when available, with an additional 8.9% of respondents reporting that they probably would not (Table 3). Conversely, 40.1% of respondents reported that they would definitely accept a free vaccine when available.

**Table 3:** Perceptions of respondents regarding the impact of COVID-19, Tennessee.

**Perception**	**Weighted frequency**	**Weighted percent**	**Weighted** 95% CI^*^
**More important impact on family**			
Economic impacts of COVID-19	4,715	48.4	45.5, 51.4
Health impacts of COVID-19	5,021	51.6	48.6, 54.5
**Concern about contracting COVID-19**			
Low/Medium	5,405	55.5	53.0, 58.0
High	4,221	43.3	40.9, 45.8
Not applicable (already infected)	114	1.2	1.0, 1.4
**Concern about own health/wellbeing**			
Low/Medium	4,863	50.1	47.0, 53.2
High	4,845	49.9	46.8, 53.0
**Concern about family’s health/wellbeing**			
Low/Medium	3,721	38.4	36.2, 40.6
High	5,978	61.6	59.5, 63.8
**Concern about financial situation**			
Low/Medium	5,241	54.0	51.7, 56.3
High	4,464	46.0	43.7, 48.3
**Concern about impact of COVID-19 on U.S.**			
Low/Medium	2,459	25.3	23.6, 27.1
High	7,266	74.7	72.9, 76.5
**Accept free COVID-19 vaccine when available**			
Probably Would Not/ Definitely Would Not	1,572	24.2	22.3, 26.2
Not Sure/ Probably Would/ Definitely Would	4,920	75.8	73.8, 77.7

**Notes.**

* CI, confidence interval.

### Predictors of opinions regarding the impact of COVID-19

All variables assessed as potential predictors had significant (*p* ≤ 0.10) univariable associations with multiple outcomes (Table 4). The final binary logistic models included between 8 and 11 predictors each (Table 5).

**Table 4:** Univariable associations between perceptions of COVID-19 impacts and their potential predictors, Tennessee.

**Characteristic**	**Health is more important impact on family than economy**		**Concern about own health/ wellbeing**		**Concern about family’s health/ wellbeing**		**Accept free COVID-19 vaccine when available**	
	OR^*^	95% CI^**^	OR^*^	95% CI^**^	OR^*^	95% CI^**^	OR^*^	95% CI^**^
**Region**								
6-County Metro Region	1.18	1.09, 1.28	1.33	1.23, 1.44	1.26	1.16, 1.37	1.08	0.96, 1.21
89-County Rural Region	–		–		–		–	
**Sex**								
Female	1.08	1.00, 1.17	1.05	0.97, 1.13	1.13	1.04, 1.23	0.61	0.55, 0.69
Male (reference)	–		–		–		–	
**Age Group**		(*p* < 0.001)		(*p* < 0.001)		(*p* < 0.001)		(*p* < 0.001)
18–24	0.67	0.58, 0.78	1.27	1.10, 1.46	1.51	1.30, 1.75	0.55	0.44, 0.69
25–44	0.62	0.55, 0.69	1.29	1.15, 1.43	1.84	1.64, 2.06	0.52	0.44, 0.62
45–64	0.55	0.49, 0.62	0.90	0.81, 1.01	1.20	1.07, 1.34	0.47	0.40, 0.56
65+ (reference)	–		–		–		–	
**Race/Ethnicity**								
White (Yes *vs.* No)	0.67	0.61, 0.74	0.46	0.41, 0.50	0.63	0.57, 0.70	1.37	1.20, 1.56
Black (Yes *vs.* No)	1.47	1.32, 1.63	2.24	2.01, 2.49	1.67	1.50, 1.87	0.66	0.58, 0.76
Asian/Pacific (Yes *vs.* No)	1.74	1.28, 2.37	2.02	1.47, 2.76	1.83	1.30, 2.57	1.75	1.06, 2.91
American Indian/Alaskan Native (Yes *vs.* No)	1.09	0.84, 1.41	1.37	1.06, 1.78	1.03	0.79, 1.35	0.83	0.59, 1.17
Hispanic/Latino, Any Race (Yes *vs.* No)	0.91	0.68, 1.21	1.55	1.16, 2.08	1.13	0.84, 1.52	0.69	0.47, 1.01
**Marital status**		(*p* = 0.003)		(*p* = 0.317)		(*p* = 0.199)		(*p* < 0.001)
Married/living with someone	1.09	0.99, 1.19	0.93	0.85, 1.02	0.99	0.90, 1.09	1.35	1.18, 1.53
Divorced/separated	1.24	1.10, 1.41	0.96	0.85, 1.09	0.90	0.79, 1.02	1.01	0.85, 1.21
Dating/single (reference)	–		–		–		–	
**Employment status**		(*p* < 0.001)		(*p* < 0.001)		(*p* < 0.001)		(*p* < 0.001)
Work full-time	0.72	0.65, 0.80	1.30	1.18, 1.45	1.58	1.42, 1.76	0.89	0.76, 1.04
Work part-time	0.81	0.70, 0.94	1.37	1.19, 1.59	1.63	1.41, 1.89	0.64	0.52, 0.78
Unemployed/looking for work	0.61	0.53, 0.70	1.51	1.32, 1.73	1.87	1.63, 2.14	0.65	0.54, 0.79
Student	0.66	0.53, 0.83	1.27	1.02, 1.59	1.41	1.12, 1.76	0.66	0.48, 0.91
At-home caregiver	0.70	0.59, 0.84	1.28	1.07, 1.52	1.53	1.28, 1.84	0.55	0.44, 0.70
Retired (reference)	–		–		–		–	
**Educational attainment**		(*p* < 0.001)		(*p* < 0.001)		(*p* < 0.001)		(*p* < 0.001)
Less than high school	1.48	1.21, 1.82	1.90	1.55, 2.32	1.73	1.39, 2.15	1.16	0.82, 1.65
High school graduate	0.92	0.83, 1.02	1.20	1.08, 1.33	1.17	1.05, 1.30	0.56	0.48, 0.65
Some college/Associate’s degree	0.80	0.73, 0.89	1.10	1.00, 1.21	1.00	0.91, 1.11	0.48	0.42, 0.56
Bachelor’s degree of higher(reference)	–		–		–		–	
**Annual household income**								
Less than $50,000	0.95	0.88, 1.03	1.26	1.17, 1.37	1.15	1.06, 1.25	0.72	0.64, 0.81
$50,000 or more (reference)	–		–		–		–	
**Household members**								
Children under age of 18 living in home (*vs.* No)	1.05	0.96, 1.14	1.40	1.28, 1.52	1.54	1.41, 1.69	0.87	0.77, 0.99
Adults over age of 50 living in home (*vs.* No)	1.25	1.15, 1.35	0.94	0.87, 1.02	0.96	0.89, 1.04	1.33	1.19, 1.49
**Infection status**								
Not previously infected	2.18	1.47, 3.23	1.70	1.15, 2.50	1.76	1.22, 2.55	1.03	0.65, 1.61
Previously infected (reference)	–		–		–		–	
**Wave**		(*p* < 0.001)		(*p* < 0.001)		(*p* < 0.001)		(*p* < 0.020)
1	0.57	0.48, 0.68	0.62	0.53, 0.74	0.74	0.63, 0.89	–	
2	0.55	0.47, 0.66	0.72	0.61, 0.86	0.83	0.70, 0.98	–	
3	0.52	0.44, 0.61	0.62	0.52, 0.74	0.77	0.65, 0.92	–	
4	0.68	0.58, 0.81	0.85	0.72, 1.01	0.94	0.79, 1.12	1.10	0.90, 1.36
5	0.58	0.49, 0.69	0.84	0.71, 0.99	0.99	0.84, 1.18	0.89	0.73, 1.09
6	0.59	0.50, 0.70	0.71	0.60, 0.84	0.78	0.66, 0.93	0.82	0.67, 1.00
7	0.74	0.62, 0.87	0.83	0.70, 0.98	0.95	0.80, 1.13	0.82	0.67, 0.99
8	0.83	0.70, 0.98	1.05	0.88, 1.24	1.13	0.94, 1.34	0.97	0.79, 1.19
9 (reference)	–		–		–		–	

**Notes.**

* OR, odds ratio.

** CI, confidence interval.

**Table 5:** Results of multivariable models used to investigate predictors of perceptions of COVID-19 impacts, Tennessee.

**Characteristic**	**Health is more important impact on family than economy**		**Concern about own health/ wellbeing**		**Concern about family’s health/ wellbeing**		**Accept free COVID-19 vaccine when available**	
	OR^*^	95% CI^**^	OR^*^	95% CI^**^	OR^*^	95% CI^**^	OR^*^	95% CI^**^
**Region**		(*p* = 0.013)		(*p* = 0.007)		(*p* = 0.003)		
6-County Metro Region	1.12	1.02, 1.22	1.13	1.03, 1.24	1.15	1.05, 1.26		
89-County Rural Region	–		–		–			
**Sex**		(*p* = 0.041)				(*p* = 0.003)		(*p* < 0.001)
Female	1.09	1.00, 1.19			1.14	1.05, 1.25	0.63	0.56, 0.71
Male (reference)	–				–		–	
**Age Group**		(*p* < 0.001)		(*p* < 0.001)		(*p* < 0.001)		(*p* < 0.001)
18–24	0.70	0.58, 0.86	0.71	0.59, 0.86	1.01	0.83, 1.23	0.62	0.49, 0.79
25–44	0.68	0.58, 0.80	0.81	0.69, 0.95	1.26	1.08, 1.47	0.53	0.44, 0.64
45–64	0.58	0.50, 0.66	0.71	0.62, 0.81	0.98	0.86, 1.12	0.46	0.38, 0.55
65+ (reference)	–		–		–		–	
**Race/Ethnicity**		(*p* < 0.001)		(*p*≤0.017)		(*p*≤0.002)		(*p*≤0.01)
White (Yes *vs.* No)	0.60	0.54, 0.67	0.65	0.53, 0.79			1.29	1.12, 1.48
Black (Yes *vs.* No)			1.41	1.15, 1.74	1.43	1.26, 1.61		
Asian/Pacific (Yes *vs.* No)			1.54	1.08, 2.19	1.74	1.23, 2.45	1.99	1.18, 3.35
American Indian/Alaskan Native (Yes *vs.* No)								
Hispanic/Latino, Any Race (Yes *vs.* No)								
**Marital status**		(*p* = 0.001)						
Married/living with someone	1.11	1.00, 1.23						
Divorced/separated	1.28	1.12, 1.46						
Dating/single (reference)	–							
**Employment status**		(*p* < 0.001)		(*p* = 0.015)		(*p* < 0.001)		
Work full-time	0.89	0.78, 1.03	1.26	1.10, 1.45	1.28	1.11, 1.47		
Work part-time	0.90	0.76, 1.06	1.21	1.02, 1.43	1.30	1.10, 1.54		
Unemployed/looking for work	0.71	0.60, 0.83	1.31	1.11, 1.54	1.40	1.19, 1.65		
Student	0.76	0.59, 0.99	1.13	0.87, 1.48	1.13	0.87, 1.48		
At-home caregiver	0.87	0.71, 1.06	1.15	0.94, 1.42	1.08	0.88, 1.33		
Retired (reference)	–		–		–			
**Educational attainment**		(*p* < 0.001)		(*p* < 0.001)		(*p* < 0.001)		(*p* < 0.001)
Less than high school	1.59	1.28, 1.97	1.63	1.31, 2.02	1.53	1.21, 1.92	1.35	0.94, 1.93
High school graduate	0.98	0.87, 1.09	1.15	1.02, 1.29	1.13	1.00, 1.28	0.62	0.52, 0.73
Some college/Associate’s degree	0.78	0.70, 0.86	1.04	0.94, 1.16	0.98	0.88, 1.09	0.51	0.44, 0.60
Bachelor’s degree of higher(reference)	–		–		–		–	
**Annual household income**				(*p* < 0.001)		(*p* = 0.012)		(*p* = 0.028)
Less than $50,000			1.23	1.12, 1.35	1.13	1.03, 1.24	0.86	0.76, 0.98
$50,000 or more (reference)			–		–		–	
**Household members**		(*p* < 0.001)		(*p* < 0.001)		(*p* < 0.001)		(*p* = 0.014)
Children under age of 18 living in home (*vs.* No)			1.36	1.23, 1.51	1.32	1.19, 1.47		
Adults over age of 50 living in home (*vs.* No)	1.19	1.08, 1.30					1.17	1.03, 1.33
**Infection status**		(*p* < 0.001)		(*p* = 0.002)		(*p* = 0.001)		
Not previously infected	2.40	1.60, 3.58	1.86	1.25, 2.77	1.90	1.30, 2.78		
Previously infected (reference)	–		–		–			
**Wave**		(*p* < 0.001)		(*p* < 0.001)		(*p* < 0.001)		(*p* = 0.008)
1	0.56	0.47, 0.67	0.59	0.49, 0.70	0.69	0.58, 0.83	–	
2	0.53	0.45, 0.63	0.68	0.57, 0.81	0.78	0.65, 0.93	–	
3	0.51	0.43, 0.61	0.58	0.49, 0.69	0.73	0.61, 0.87	–	
4	0.67	0.56, 0.79	0.81	0.68, 0.97	0.91	0.76, 1.09	1.21	0.98, 1.49
5	0.57	0.48, 0.68	0.78	0.66, 0.93	0.95	0.79, 1.13	0.95	0.77, 1.16
6	0.57	0.47, 0.67	0.68	0.57, 0.81	0.76	0.64, 0.91	0.85	0.69, 1.04
7	0.73	0.61, 0.86	0.80	0.68, 0.95	0.93	0.78, 1.11	0.85	0.70, 1.04
8	0.82	0.69, 0.97	1.02	0.85, 1.21	1.11	0.92, 1.32	1.02	0.83, 1.26
9 (reference)	–		–		–		–	

**Notes.**

* OR, odds ratio.

** CI, confidence interval.

#### Model 1: Health or economic impact on family is more important

Predictors of the perception that COVID-19 had a more important impact on the health of their family included region, sex, age group, White race, marital status, employment status, educational attainment, adults over age 50 in the household, infection status, and study wave (Table 5). Compared to respondents from rural counties, those from metropolitan counties had higher odds (OR = 1.12, *p* = 0.013) of reporting that the health impact was greater. The odds were slightly higher for females compared to males (OR = 1.09, *p* = 0.041). Age group was also a significant (*p* < 0.001) predictor, with lower odds of reporting health as the greater impact on their family at all age categories compared to those age 65 and above. Similarly, respondents who reported their race as White had lower odds (OR = 0.60, *p* < 0.001) of reporting health as the greater impact than those that did not self-identify as White, whether alone or in combination.

Model 1 was the only model for which marital status was identified as a significant (*p* < 0.001) predictor, with married/partnered respondents and divorced/separated respondents having higher odds (OR = 1.11, *p* = 0.050, and OR = 1.28, *p* < 0.001, respectively) of reporting health as the greater impact compared to those respondents who were dating/single. Employment status was a significant (*p* < 0.001) predictor as well, with all employment categories having lower odds (range: OR = 0.71–0.90) of reporting health as the greater impact, compared to respondents who were retired. Educational attainment was a significant (*p* < 0.001) predictor but played a variable role. Whereas respondents with less than a high school education had higher odds (OR = 1.59, *p* < 0.001) of reporting health as the greater impact compared to those with a bachelor’s degree or higher, those who had attended some college had significantly lower odds (OR = 0.78, *p* < 0.001) in comparison to those with a bachelor’s degree or higher.

Household makeup played a notable role in the model, with respondents who reported adults over age 50 in the home having higher odds (OR = 1.19, *p* < 0.001) of reporting health as the greater impact than those respondents without an older adult in the home. Respondents who had not been previously infected with COVID-19 had much higher odds (OR = 2.40, *p* < 0.001) of reporting health as the greater impact on their family compared to those respondents that had been previously infected. Finally, survey wave was a significant (*p* < 0.001) predictor of whether respondents would report the health or economic impact as greater, with respondents in all survey waves from May through November 2020 having lower odds (range: OR = 0.51–0.82) of reporting health as the more important impact compared with respondents in the December 2020 wave.

#### Model 2: Concern about own health and wellbeing

Predictors of high level of concern about respondent’s health and wellbeing included region, age group, White race, Black race, Asian race, employment status, educational attainment, annual household income, children under age 18 in the household, infection status, and study wave (Table 5). Compared to respondents from rural counties, those from metropolitan counties had higher odds (OR = 1.13, *p* = 0.007) of reporting a high level of concern about their own health and wellbeing. Age group was a significant (*p* < 0.001) predictor, with lower odds of reporting a high level of concern about their health and wellbeing at all age categories compared to those age 65 and above. White respondents had lower odds (OR = 0.65, *p* < 0.001) of reporting a high level of concern about their health and wellbeing than non-White respondents, while Black or Asian respondents had higher odds (OR = 1.41, *p* = 0.001, and OR = 1.54, *p* = 0.017, respectively) than those that were non-Black or non-Asian.

Respondents in all employment categories had higher odds (range: OR = 1.13–1.31) of having a high level of concern about their own health, compared to respondents who were retired. Respondents with less than a high school education and those with a high school degree had higher odds (OR = 1.63, *p* < 0.001, and OR = 1.15, *p* = 0.022, respectively) of reporting a high level of concern about their health and wellbeing compared to those with a bachelor’s degree or higher. Compared with respondents reporting an annual household income of ≥$50,000, those with annual household income of <$50,000 had higher odds (OR = 1.23, *p* < 0.001) of reporting a high level of concern about their health. Respondents that reported children under 18 years old living in the home had higher odds (OR = 1.36, *p* < 0.001) of reporting a high level of concern about their health than those without children in the home. Respondents who had not been previously infected with COVID-19 had higher odds (OR = 1.86, *p* = 0.002) of reporting a high level of concern about their health compared to those that had been previously infected.

#### Model 3: Concern about family’s health and wellbeing

Predictors of respondents’ concern about their family’s health and wellbeing included region, sex, age group, Black race, Asian race, employment status, educational attainment, annual household income, children under age 18 in the household, infection status, and study wave (Table 5). Compared to respondents from rural counties, those from metropolitan counties had higher odds (OR = 1.15, *p* = 0.003) of reporting a high level of concern about their family’s health and wellbeing. The odds of high level of concern about respondent’s family’s health were higher among female respondents than their male counterparts (OR = 1.14, *p* = 0.003). Only respondents aged 25–44 years had higher odds of reporting a high level of concern about their family’s health compared to those ≥65 years. Black or Asian respondents had higher odds (OR = 1.43, *p* < 0.001, and OR = 1.74, *p* = 0.002, respectively) of reporting a high level of concern about their family’s health and wellbeing than non-Black or non-Asian respondents.

Respondents in all employment categories had higher odds (range: OR = 1.08–1.40) compared to those who were retired. Respondents with less than a high school education and those with a high school degree had higher odds (OR = 1.53, *p* < 0.001, and OR = 1.13, *p* = 0.039, respectively) of reporting a high level of concern about their family’s health and wellbeing compared to those with a bachelor’s degree or higher. Compared to respondents reporting an annual household income of ≥$50,000, those with annual household income of <$50,000 had higher odds (OR = 1.13, *p* = 0.012) of reporting a high level of concern about their family’s health. Respondents that reported children under age 18 years living in the home had higher odds (OR = 1.32, *p* < 0.001) of reporting a high level of concern about their family’s health than those without children in the home. Respondents who had not been previously infected with COVID-19 had higher odds (OR = 1.90, *p* = 0.001) of reporting a high level of concern about their family’s health compared to those that had been previously infected.

#### Model 4: Willingness to accept a free COVID-19 vaccine when available

Predictors of respondents reporting that they would accept a free COVID-19 vaccine included sex, age group, White race, Asian race, educational attainment, annual household income, adults over age 50 in the household, and study wave (Table 5). In contrast to the previous models for which sex was a significant predictor, in which females had higher odds, in this model, female respondents had much lower odds (OR = 0.63, *p* < 0.001) of reporting vaccine acceptance than males. Respondents in all age categories had lower odds (range: OR = 0.46–0.62) of reporting that they would accept a free COVID-19 vaccine compared to those age ≥65 years. Also, in contrast to previous models in which White respondents had lower odds of expressing concerns, in this model White respondents had higher odds (OR = 1.29, *p* < 0.001) of reporting that they would accept a free COVID-19 vaccine once available than non-White respondents. Asian respondents had odds twice as high as non-Asian respondents (OR = 1.99, *p* = 0.010) of reporting that they would accept a free COVID-19 vaccine.

Respondents with less than a high school education had higher odds (OR = 1.35, *p* = 0.105) of willingness to accept a free COVID-19 vaccine compared to those with a bachelor’s degree or higher, while those who had a high school degree or had attended some college had significantly lower odds (OR = 0.62, *p* = 0.001, and OR = 0.51, *p* < 0.001). Compared with respondents reporting an annual household income of ≥$50,000, those with annual household income of <$50,000 had lower odds (OR = 0.86, *p* = 0.028) of reporting that they would accept a free COVID-19 vaccine once available. Respondents that reported adults over age 50 living in the home had higher odds (OR = 1.17, *p* = 0.014) of willingness to accept a COVID-19 vaccine than those respondents without older adults in the home.

## Discussion

This study investigated geographic, socioeconomic, and demographic differences in the opinions of Tennessee residents regarding the impacts of COVID-19 as well as predictors of these opinions. Improving our understanding of the population differences in opinions and factors which drive these differences may help public health authorities more effectively address public concerns about the pandemic and may be useful in guiding response options.

### Descriptive characteristics

Respondents’ opinions regarding the relative importance of the economic or health impact of COVID-19 on their families were in contrast to those of a national survey of adults, which reported that 34% identified the economic impact of the pandemic as a major problem in their community, while 26% identified the health impact as major problem (Parker, Horowitz & Minkin, 2021). Whereas just under half of respondents in the current study reported high levels of concern for their own health and almost two-thirds reported high levels of concern for the health of their family members, other studies have also reported higher levels of concern for family members than for respondents’ own health (Barber & Kim, 2021). The low reported intention to definitely accept a COVID-19 vaccine (40%) is consistent with reports from other studies of vaccine hesitancy in Tennessee (Alcendor, 2021) and elsewhere in the United States (Guidry et al., 2021; Khubchandani et al., 2021; Lindholt et al., 2021), but contrasts with reported high intention to accept in low- and middle-income countries (Joshi et al., 2021; Moola et al., 2021; Wang et al., 2021).

#### Predictors of relative importance of health or economic impact on family

Respondents were nearly evenly divided regarding whether the health or economic impact of COVID-19 was greater for their families, which is in contrast to the reported findings of a national survey in which a larger percentage of the population felt that the economic impact of COVID-19 was a major problem (Parker, Horowitz & Minkin, 2021). Whereas in Tennessee the residents of metro areas had higher odds of reporting that the health impact of COVID-19 was greater than the economic impact (OR = 1.12, *p* = 0.013), a study in Indiana found that residents of metro areas reported greater economic precarity as a result of COVID-19 (Perry, Aronson & Pescosolido, 2021). Yet a nationwide study of the impact of COVID-19 on rural areas identified a greater negative perception of the impact on household finances than physical health (Mueller et al., 2020). The observed significant association in the present study between region and reporting the health impact of COVID-19 to be greater might be due to the fact that in Tennessee, the more economically disadvantaged rural areas are both older and largely White, with each of these demographic categories reporting differing concerns. Indeed, White individuals were significantly less likely to report that the health impact was more important. This is consistent with the Pew Research Center report which identified White adults as least likely to report the health impact of COVID-19 as a major problem in their community (Parker, Horowitz & Minkin, 2021).

This study found that respondents with less than a high school education had higher odds (OR = 1.59, *p* < 0.001) of reporting that the health impact was greater on their families than the economic impact, which appears to contrast the association between educational attainment and economic precarity reported by Perry et al. in Indiana (Perry, Aronson & Pescosolido, 2021). Likewise, a nationally representative survey of the financial impact of COVID-19 reported that educational attainment of less than a bachelor’s degree was a significant predictor of financial vulnerability (Bruce et al., 2022). However, neither study assessed the relative contrast of health *versus* economic impact. It may be that the findings of the present study reflect a greater likelihood among respondents with lower educational attainment to work in positions which have been designated “essential,” thereby ensuring their continued employment, but also placing them at potentially greater risk of infection.

### Predictors of concerns about health and wellbeing

The results of this study indicate that the pandemic has also had a significant impact on the wellbeing of Tennesseans. Predictors of respondents’ concern for their own health and wellbeing included region, age group, educational attainment and race. Odds of reporting concern for their own health were significantly higher among Black (OR = 1.41) and Asian (OR = 1.54) respondents than their non-Black and non-Asian counterparts, respectively. This contrasts with findings from a study by Kämpfen et al. which reported greater mental health concerns being associated with COVID-19 infections among White respondents. The authors of that study proposed that this may reflect increased economic pressure among non-White communities (Kämpfen et al., 2020). Indeed, such an understanding of the economic role may also be interpreted in the present study, in which respondents reporting household income below $50,000 had significantly higher odds of reporting concern for their health.

In the present study, all employment categories had higher levels of concern compared to retirees, yet younger respondents had lower odds of being concerned about their own health than those 65 years and older. These seemingly contradictory findings are consistent with reports from other studies of COVID-19 perceptions conducted in the early phases of the pandemic (Whatley et al., 2020; Barber & Kim, 2021). For instance, a large US study reported that older adults perceived themselves to be at lower risk of COVID-19 infection, but at greater risk of experiencing a severe outcome if infected (De Bruin, 2021). Another study reported that older adults perceived COVID-19 to present a greater general risk than did younger adults, while the concern over either personally catching or dying from the disease demonstrated significant interaction between age and sex (Barber & Kim, 2021). The findings of the present study may reflect the ability of older respondents to remain at home and observe social distancing more effectively, as well as concerns about more serious illness if infected, due to waning immunity and pre-existing conditions among the older adults.

Respondents living in households with children under age 18 had higher odds of reporting concern for their own health than those without children. However, it is unclear whether the reported concerns are due to worries that children were likely to be the sources of infection for the respondents or that if the respondents were to become ill, they would be unable to care for the children. The latter coincides with the reported findings of an Australian study of public perceptions in which respondents expressed concerns about the potential impact of isolation on caregiving responsibilities (Seale et al., 2020). In comparison to older adults in the present study, only respondents aged 25–44—the age group with the highest percentage of children in the household—had significantly higher odds (OR = 1.26) of reporting concern for the health of their family members. A U.K. study reported that rates of compliance with pandemic guidelines was lower among adults living with a child (Wright & Fancourt, 2021). The study authors attributed this to diminishing resource availability that placed individuals of this age group under greater economic, physical, and psychological stress during the pandemic. The present study’s finding of higher odds of concern for family’s health among the middle-aged individuals is therefore important in terms of both the mental health of this group and the economic pressures which may drive health behaviors. These findings are also noteworthy given that a US study reported that the percentage of households with children was a significant positive predictor of both county-level COVID-19 incidence and mortality (Karmakar, Lantz & Tipirneni, 2021).

### Predictors of willingness to accept a free COVID-19 vaccine when available

In the present study, approximately three-quarters (75.8%) of respondents indicated that they would possibly or likely accept a free COVID-19 vaccine, which is consistent with national assessments of vaccine acceptance prior to the broad availability of COVID vaccines (Khubchandani et al., 2021; Mondal, Sinharoy & Su, 2021). The reported levels of vaccine hesitancy appear to have been borne out in Tennessee, which ranked in the lowest quintile of states for complete vaccination during 2021 (Mayo Clinic, 2022). Yet vaccine hesitancy appears unevenly distributed across the population with sex, age, education, income, and household composition being identified as predictors of willingness to accept a free COVID-19 vaccine. In the present study, women were less likely to accept a vaccine, which is consistent with both national (Guidry et al., 2021; Mondal, Sinharoy & Su, 2021) and international (Joshi et al., 2021; Karlsson et al., 2021; Paul, Steptoe & Fancourt, 2021; Wang et al., 2021; Yan, Lai & Lee, 2021) survey results. However, in contrast to the finding in this study that lower educational attainment positively predicted vaccine acceptance, other nationwide studies have reported greater vaccine acceptance among individuals with a bachelor’s degree or higher (Guidry et al., 2021; Mondal, Sinharoy & Su, 2021). White and Asian respondents had significantly higher odds (OR = 1.29 and OR = 1.99, respectively) of willingness to be vaccinated than other races, which agrees with the findings reported by previous surveys across the US (Guidry et al., 2021; Khubchandani et al., 2021; Mondal, Sinharoy & Su, 2021).

### Strengths and weaknesses

A key strength of this study is the use of prospectively collected survey data and rigorous statistical analyses to investigate predictors of opinions influencing health behaviors related to COVID-19. Although several studies have investigated predictors of COVID-19 vaccine acceptance among both broad and local populations, few have addressed predictors of opinions influencing other health behaviors. Therefore, this study represents a unique contribution to our understanding of the factors which play a role in the perceptions of the population. Yet, this study is not without limitations. Survey data regarding the perceptions around COVID-19 were self-reported and may therefore be subject to reporting bias. Nonetheless, the findings of this study may help identify key factors influencing health behaviors and guide development of strategies to overcome vaccine hesitancy in states with a mixed rural/urban population.

#### Conclusions

This study found geographic, demographic, and temporal differences with regard to perceptions of COVID-19 and the intention to accept COVID-19 vaccine. The findings of this study are beneficial for public health administrators and may be useful in understanding drivers of health behaviors and developing strategies to address vaccine hesitancy.

##  Supplemental Information

10.7717/peerj.15473/supp-1 Supplemental Information 1 Study Data

## List of Abbreviations

COVID: Coronavirus Disease
IRB: Institutional Review Board
OR: Odds Ratio
SARS-CoV-2: Severe Acute Respiratory Syndrome Coronavirus 2
TN: Tennessee
US: United States
WHO: World Health Organization

## Additional Information and Declarations

### Data Availability

The following information was supplied regarding data availability: The study data is available in the Supplemental Files.
